# Intrasexual competition facilitates the evolution of alternative mating strategies in a colour polymorphic fish

**DOI:** 10.1186/1471-2148-10-391

**Published:** 2010-12-23

**Authors:** Jorge L Hurtado-Gonzales, J Albert C Uy

**Affiliations:** 1 Life Sciences Complex 234, Department of Biology, Syracuse University, Syracuse, NY 13244, USA; 2 Department of Biology, University of Miami, Coral Gables, FL 33124, USA

## Abstract

**Background:**

Intense competition for access to females can lead to males exploiting different components of sexual selection, and result in the evolution of alternative mating strategies (AMSs). Males of *Poecilia parae*, a colour polymorphic fish, exhibit five distinct phenotypes: drab-coloured (immaculata), striped (parae), structural-coloured (blue) and carotenoid-based red and yellow morphs. Previous work indicates that immaculata males employ a sneaker strategy, whereas the red and yellow morphs exploit female preferences for carotenoid-based colours. Mating strategies favouring the maintenance of the other morphs remain to be determined. Here, we report the role of agonistic male-male interactions in influencing female mating preferences and male mating success, and in facilitating the evolution of AMSs.

**Results:**

Our study reveals variation in aggressiveness among *P. parae *morphs during indirect and direct interactions with sexually receptive females. Two morphs, parae and yellow, use aggression to enhance their mating success (i.e., number of copulations) by 1) directly monopolizing access to females, and 2) modifying female preferences after winning agonistic encounters. Conversely, we found that the success of the drab-coloured immaculata morph, which specializes in a sneak copulation strategy, relies in its ability to circumvent both male aggression and female choice when facing all but yellow males.

**Conclusions:**

Strong directional selection is expected to deplete genetic variation, yet many species show striking genetically-based polymorphisms. Most studies evoke frequency dependent selection to explain the persistence of such variation. Consistent with a growing body of evidence, our findings suggest that a complex form of balancing selection may alternatively explain the evolution and maintenance of AMSs in a colour polymorphic fish. In particular, this study demonstrates that intrasexual competition results in phenotypically distinct males exhibiting clear differences in their levels of aggression to exclude potential sexual rivals. By being dominant, the more aggressive males are able to circumvent female mating preferences for attractive males, whereas another male type incorporates subordinate behaviours that allow them to circumvent male aggression and female mating preferences. Together, these and previous results indicate that exploiting different aspects of social interactions may allow males to evolve distinct mating strategies and thus the long term maintenance of polymorphisms within populations.

## Background

Intense sexual selection can lead to competing males evolving alternative ways to obtain fertilizations, thereby enhancing their reproductive success [1]. In genetically-based polymorphic species, alternative mating strategies (AMSs) are characterized by distinct behavioural and morphological traits that help males (hereafter morphs) ameliorate their mating disadvantages when facing superior competitors [2-4]. For example, in some polymorphic lizards [5,6], birds [7-9], fish [10,11] and marine isopods [12], large and/or colourful males are aggressive and defend breeding territories to exclude competitors. As a consequence, subordinate males have evolved AMSs, such as sneak copulations that are accompanied by adaptations to sperm competition [1] to possibly circumvent overt aggression. AMSs are expected to be maintained as long as the resulting average fitness of one strategy equals that of the others co-occurring in the population [4], with frequency-dependent selection favouring rare over common phenotypes [2,4,13].

Sexual selection theory predicts that strong mating preferences for males with elaborate ornaments that reflect their quality or dominance should deplete genetic variation in these traits [14-17]. There are, however, cases in which males as a result of high variance in mating success and thus strong sexual selection exhibit striking, genetically-based polymorphism in display traits (e.g., [10,18-20]). Further, several studies in a broad range of taxa ([5,7-10,12,21-30] see also Table 12.2 in [4]) consistently suggest that such variation in male phenotypes (e.g., extreme differences in body lengths, behaviours, physiology) is adaptive and correlates with asymmetric social dominance relationships defining AMSs. A mechanism by which variation in male phenotypes is maintained is through frequency dependent selection for AMSs [2,4,12,13]. For instance, in the side-blotched lizard (*Uta stansburiana*), males have evolved AMSs (orange-throated: aggressive and territorial; yellow-throated: sneaker; blue-throated: mate guarding) and the relative fitness of each strategy fluctuates depending on the frequency of the competing strategies from one generation to the next [5]. Under frequency-dependent selection, the rare strategy appears to have mating advantage [2,31]. Negative frequency-dependent selection is often invoked to explain polymorphisms that are not shaped by sexual selection [32-36].

Although not necessarily independent of frequency dependent selection, a less explored mechanism for the maintenance of polymorphisms involves complex, balancing selection in which different aspects of sexual and natural selection select for unique phenotypes (e.g., [13]). For instance, female mating preferences may favour colourful males, while agonistic male-male competition may favor large males [10]. This lack of synergism between the two aspects of sexual selection may allow for the invasion of AMSs and thus promote the maintenance of polymorphisms [10,18-20,22].

In many species, males can use overt aggression as a tactic to circumvent female choice [37-39]. For instance, dominant males can prevent attractive males from gaining access to females [10,40-43]. However, male aggression can also facilitate female choice if winners of male contests signal their quality (e.g., vigour, tenancy of better territories) to females [37-39]. Further, in many taxa, the success of mating attempts ultimately relies on female consent [44], and thus; females may still exercise choice despite overt male aggression [41,42]. This potential for conflict and synergism between intrasexual competition and intersexual mate choice can result in opportunities for certain males to exploit different components of sexual selection [3], facilitating the evolution and maintenance of AMSs [4,44,45]. Here, we explore how overt male aggression can influence male mating success and facilitate the persistence of colour polymorphism in the pentamorphic fish *Poecilia parae*.

The South American poeciliid *P. parae *exhibit five Y-linked, discrete colour morphs [46,47]. These morphs include: (i) immaculata, the smallest and drab-coloured males that resemble juvenile females; (ii) parae, the largest males that exhibit a striped tail and black vertical body bars that intensify during social interactions; and (iii) the blue, red, and yellow males that are of intermediate body size and display colourful body flanks [46,48]. Males and females mate promiscuously, with males providing no resources during mating [48]. *Poecilia parae *breeds year-round [48,49], suggesting an opportunity for intense competition among males to identify and monopolize sexually receptive females. In *P. parae*, the carotenoid-based red and yellow morphs are strongly preferred by the majority of females as mates, and the smaller immaculata males are the least attractive males [46,49,50]. However, because the immaculata morph specializes in sneak copulations with apparent adaptations for sperm competition [51], such a mating strategy would be successful only if sneaker males can circumvent pre-copulatory female choice, intrasexual aggression, or both (e.g.,[4,5,7]. It remains to be seen, however, whether immaculata males can indeed circumvent male aggression and female choice and consequently gain successful matings. The persistence of the other two morphs (*i.e*., parae and blue) may be the result of their competitive abilities in open mixed groups [46,49].

In this study, we experimentally test for the role of male-male competition in the evolution and maintenance of AMSs. If indeed, particular morphs specialize in male-male aggression, we predict that agonistic interactions (i) may limit the mating opportunities of the attractive (i.e., red and/or yellow) morphs, (ii) have the potential to influence female mating preferences, and (iii) enhance the mating success of aggressive males. Results consistent with these predictions would suggest that the opportunity to exploit male-male competition may facilitate the maintenance of genetically-based polymorphisms.

## Results

### (a) Female mate choice and male dominance

**Figure 1:**
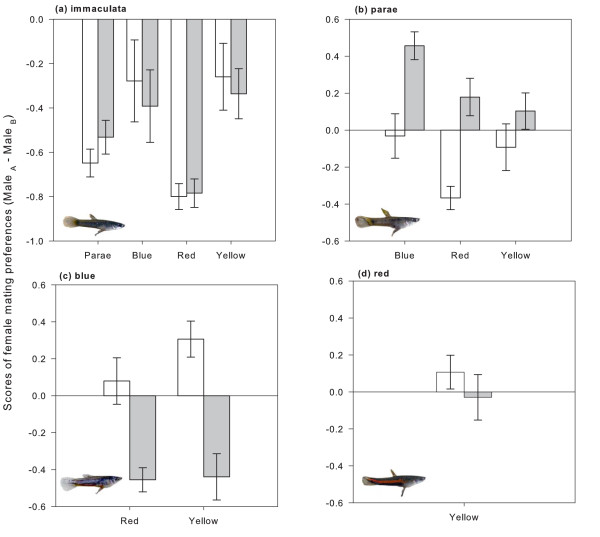
**Mean (± standard error) female mate choice scores during pre- (empty bars) and post- male competition (filled bars) trials for immaculata, parae, blue and red morphs**. Positive results indicate female preference for pictured males (i.e., Male A) while negative results indicate preference for the assigned opponents (i.e., Male B, or males in X-axis).

On average, females spent 30.81 ± 15.67% of the time in the region close to the test males. In the experiment where males were not allowed to interact (pre-male competition), females spent more time with the parae, blue, red and yellow than with the drab immaculata males (Figure 1a). After observing male-male interactions (post-male competition), females switched their mating preferences when parae males were winners of interactions against blue (paired t_14 _= P < 0.01), red (paired t_14 _= P < 0.01), and yellow males (paired t_14 _= P = 0.1; Figure 1b). Similarly, aggression displayed towards the blue males modified female choice favouring red (paired t_14 _= P < 0.01) and yellow (paired t_14 _= P < 0.01) males (Figure 1c). We found no statistical differences between the mate choice scores of red and yellow males (paired t_14 _= P = 0.23) for the pre-male and post-male competition experiments (Figure 1d).

On average, parae males dominated blue, red and yellow males (binomial tests, all P < 0.01). Immaculata males never initiated aggressive interactions during the staged contests against other males, and they were attacked most frequently by yellow males (Mean ± SE: 12 ± 3.41 aggressive behaviours 10 min^-1^) and in less proportion by parae males (2.7 ± 0.83 aggressive behaviours 10 min^-1^). Blue males typically lost to red (binomial test, P = 0.01; n = 15) and yellow (binomial test, P < 0.01; n = 15) males. Yellow males were dominant in 60% of their encounters with red males (binomial test, P = 0.30; n = 15).

**Figure 2:**
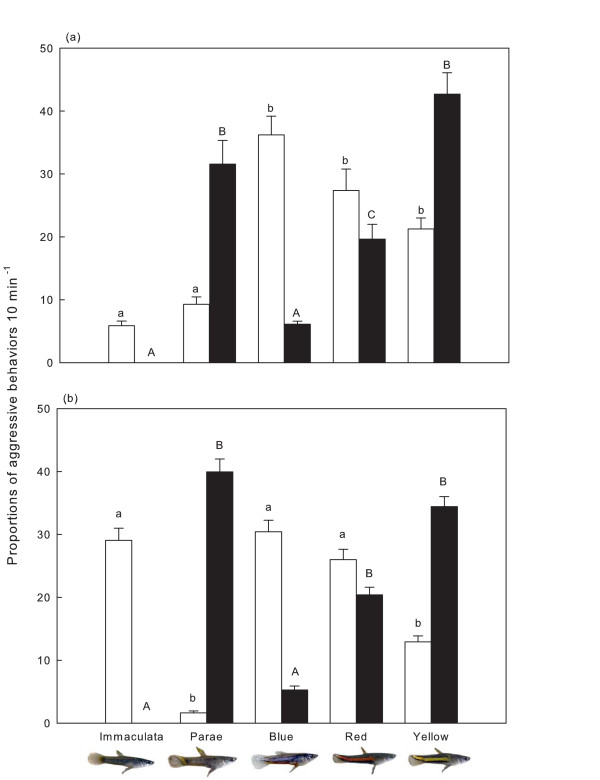
**Mean (± standard error) proportions of received (empty bars) and initiated (filled bars) aggressive behaviours 10 min^-1 ^for each morph when (a) females were separated but were able to observe male-male interactions, and (b) females freely interacted with competing males in open aquaria**. Bars with different letters above are significantly different (see text).

In addition, the five morphs differed in the rate of received (Kruskal-Wallis test: H_4, 260_: 37. 55, P < 0.01) and initiated (Kruskal-Wallis test: H_4, 260_: 74. 03, P < 0.01) aggressive behaviours (Figure 2a). Overall, the immaculata and parae morphs were less likely to be attacked when compared to the blue, red, and yellow males (all comparisons, P < 0.01). In contrast, parae males initiated more aggressive behaviours than the immaculata, blue and red morphs (all comparisons, P < 0.01). Parae and yellow males did not differ significantly in rate of initiating aggression (P = 0.09).

### (b) Competition for access to females and mating success

The total number of aggressive behaviours was fewer when males were allowed to directly interact with females than when females were separated from males and only observed male-male interactions (direct: Mean ± SE: 7.12 ± 1.07 aggressions 10 min^-1^; n = 220; indirect: 25.85 ± 2.91 aggressions 10 min^-1^; n = 220; Mann-Whitney U = 147, P < 0.01). Morphs differed in the number of aggressive behaviours received (Kruskal-Wallis test: H_4, 260_: 35. 09, P < 0.01) and initiated (Kruskal-Wallis test: H_4, 260_: 56. 74, P < 0.01; Figure 2b) during the open aquaria experiment. Parae and yellow males received fewer attacks (P < 0.01), but, along with red males, initiated the greater proportion of aggressive behaviours (P < 0.02). Compared to parae, blue, and red males, yellow males initiated more aggressive behaviours (Mean ± SE: 23.70 ± 4.18; n = 10; 2% sparring, 53% chasing, and 45% attacks) against immaculata males. In contrast, parae males initiated the least number of aggressive behaviours towards immaculata males (Mean ± SE: 1 ± 0.52 aggressive behaviours 10 min^-1^), yet directed more attacks to blue (Mean ± SE: 24.13 ± 7.59; n = 15) and red (14.6 ± 4.46; n = 15) males.

**Figure 3:**
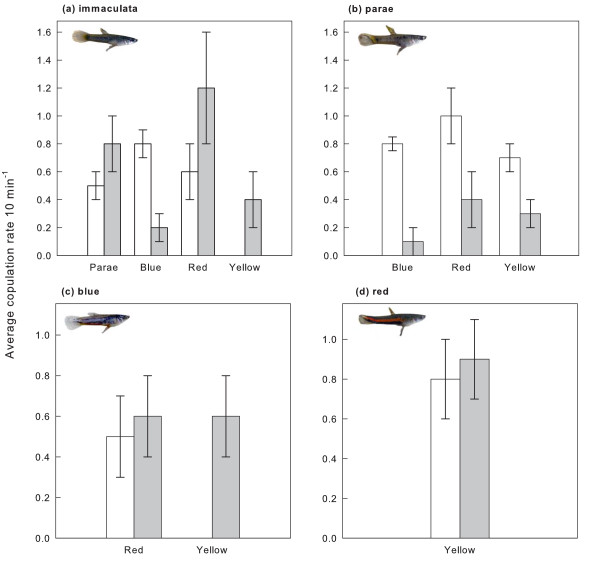
**Mean (± standard error) rates (10 min^-1^) of gained (empty bars) and lost (filled bar) copulations by immaculata, parae, blue and red morphs (in pictures) against assigned opponents in the experiment where females could physically interact with competing males**.

The number of copulations (Mean ± SE: 0.56 ± 0.07 10 min^-1^) gained as a result of individuals being able to fend off their competitors differed significantly among morphs (Kruskal-Wallis test: H_4, 260_: 28.31, P < 0.01; Figure 3). Parae males experienced an increase in mating success when competing against blue (Wilcoxon matched paired test, z = 2.69, n = 15, P < 0.01) and red (Wilcoxon matched paired test, z = 2.2, n = 15, P = 0.02; Figure 3) males, but not with yellow males (Wilcoxon matched paired test, z = 1.53, n = 15, P = 0.13).

Yellow males obtained significantly more copulations after winning against blue males (Wilcoxon matched paired test, z = 2.67, n = 15, P < 0.01) and marginally significant after winning against immaculata males (Wilcoxon matched paired test, z = 1.83, n = 10, P = 0.06). Moreover, 11.67% of copulations obtained by yellow males were the result of aggressive interactions (*i.e*., sparring) with females and sneak copulations.

## Discussion

Our results suggest that certain *P. parae *morphs have evolved the use of overt aggression as an AMS. Several lines of evidence support this interpretation. First, males of the parae and yellow morphs consistently dominated their competitors during staged contests by excluding rival males from gaining access to females. Second, when females and competing males were allowed to freely interact, parae and yellow males gained a substantial proportion of matings by directly monopolizing females and limiting the mating opportunities of the blue and red morphs, explicitly showing that aggression indeed results in increased mating success for dominant males. Finally, females observing male-male interactions modified their mate choice favouring dominant over subordinate males for contests that involved males of the parae, red, and yellow morphs. These results suggest that the opportunity for intense intrasexual competition can facilitate the evolution and maintenance of AMSs when females display strong preferences for attractive but not necessarily dominant males (see also [10,40,43]).

During the staged contests, immaculata males, the smallest and the least preferred males by females, were typically submissive. In our study, the mating success of immaculata males relied on the strategy of appearing like juvenile females to avoid harassment when approaching receptive females and in taking advantage of their smaller body size to sneak copulations. The juvenile female-mimic strategy was mostly effective when immaculata males were competing with the parae, blue, and red males. These behavioural patterns suggest that the immaculata morph has evolved a strategy to circumvent male agonistic interactions and female mate choice (e.g., [5,52,53]).

Our experiments are consistent with previous findings that uncovered variation in female mating preferences for colourful males [46,49,50]. When male-male competition is excluded, a large proportion of females strongly preferred red and yellow males; however, some females showed consistent preferences for parae and blue males as well [50]. In the experiments where females were physically separated from males with a clear barrier, red and yellow males did not differ in their ability to attract females, even after the test females observed yellow males dominate over red males. However, in the experiments where males and females could physically interact, yellow males were dominant over red males and were capable of restricting access of red males to females. This difference in aggression translated in greater association time with females for yellow males. Surprisingly, however, the observed preference did not differ in their realized mating success (i.e., number of copulations in 10 min^-1^), suggesting that even though aggressive yellow males can restrict the access of red males to females, female preference for red males was able to counteract male aggression. That is, in cases in which red males were subordinate, females still managed to circumvent the attempts of dominant yellow males to monopolize matings and mated with the attractive red males. Similar female mating behaviours have also been reported in guppies (*P. reticulata*), wherein paternity in multiply sired broods was biased towards subordinate males ([40], but see also [54]).

How then does intrasexual competition contribute to the maintenance of the striking colour polymorphism in *P. parae*? Because sexual selection operates at distinct stages (e.g., pre- and postmating intersexual choice, pre- and postmating intrasexual competition), males can evolve unique strategies that specialize in one or few stages of sexual selection (reviewed in [3]). For instance, females may have strong mating preferences for particular males, but the most dominant and aggressive males can exclude attractive males from gaining access to females [37-39]. Other AMSs can circumvent both female choice and male aggression by mimicking females and adopting a sneaker strategy (e.g., yellow sneaker in side-blotched lizard; beta and gamma males in marine isopods; satellite and 'faeders' in the ruff [5,52,53]). Furthermore, males exploiting postcopulatory sexual selection could also evolve sperm related traits that enhance their competitive abilities during sperm competition (e.g., allocating ejaculates containing faster sperm; see [3]) or postcopulatory female choice (e.g., [55]).

Indeed, several studies have demonstrated that under intense episodes of sexual selection, males experiencing continuous mating disadvantages should evolve strategies that exploit different components of sexual selection (e.g., the ruff, [7]; side-blotched lizard: [56]; pigmy swordtail [10]; Gouldian finch, [8]; marine isopods, [53]). In these examples, frequency-dependent selection is the most plausible explanation in facilitating the co-existence of the AMSs [2,4]. In *P. parae*, however, frequency-dependent selection cannot fully explain the persistence of the five morphs, as their frequency in nature is consistent over time ([46,48]; see Figure 2. in [50]). Field surveys from 2002 to 2009 indicate that immaculata and parae males are the most abundant, followed by blue males, with red and yellow males being rarest [50]. In this case, different forms of balancing selection may help explain the persistence of the five morphs in *P. parae*. For instance, females show a strong mating preference for the red and yellow males [46,49,50], and so these males should be most abundant. Red and yellow males, however, are rare, and this may be due to strong predator (i.e., cichlids) preference for prey with carotenoid colour patches [50]. Therefore, the interaction between female mating preference and selective predation may provide opportunities for less attractive males to evolve AMSs, and invade and persist in the population. Our study suggests that the parae (and to some extent also yellow) morph use intrasexual aggression to monopolize females and obtain matings. However, in this and many other systems, female cooperation is needed to achieve successful matings (e.g. [44]), and so, while parae males are able to monopolize access to females, female mate choice may still counteract the effects of male aggression, and prevent parae and yellow males from driving the other morphs to extinction. The drab female-mimic immaculata morph, besides being cryptic to visual predators, represents an alternative strategy that efficiently seems to circumvent both female mating preferences and male-male competition (see also [46]). This form of balancing selection in which female mate choice and intrasexual competition, combined with predator preferences for red and yellow males, interact may ultimately provide opportunities for AMSs to evolve and persist in the population.

Lastly, although blue males gained some matings as a result of aggressive interactions and variable female mating preferences [50], the factors that allow for the persistence of this morph is under current analysis. Recent studies suggest a strong role for environmental heterogeneity in favouring the maintenance of colour polymorphisms [57-59]. For instance, as shown in the pentamorphic Sulawesi fish, *Telmatherina sarasinorum *[59], female mating preferences may vary as a response to spatial and/or temporal fluctuations in the visual environment [57]. Preliminary surveys of *P. parae *habitat indicate that the visual habitat is variable, with some areas being rich in short wavelength light. This suggests the intriguing possibility that, in their natural settings, blue males may be able to exploit microhabitats rich in short wavelengths ambient light to appear more conspicuous and thus more attractive to females (Hurtado-Gonzales & Uy; in prep). Hence, the role of sexual selection under environmental heterogeneity may explain the persistence of the blue *P. parae *morph.

## Conclusions

Frequency dependent selection is typically invoked to explain the evolution and maintenance of genetically-based polymorphisms/AMSs. Under this scenario, morphs should experience cyclical fluctuation in their frequencies, with rare morphs having a selective advantage over common morphs. Alternatively, and somewhat independent of frequency dependent selection, complex balancing selection may explain the evolution and maintenance of polymorphisms in cases where the frequencies of morphs experience little or no changes over time. In our work, the antagonistic interaction between different components of sexual selection (i.e., pre- and postcopulatory sexual selection), in addition to natural selection by predators, allows for the invasion of AMSs and thus the maintenance of the striking polymorphism in *P. parae*.

## Methods

Female mating preferences and male aggression were assessed using wild caught fish from the west coast of the Demerara River (6° 41' N, 58° 12' W), Republic of Guyana. Males were sorted by morph type (n = 40 immaculata; n = 55 parae; n = 55 blue; n = 38 red; and n = 41 yellow) and housed in separate aquaria with non-experimental females at equal sex ratio. Fish were maintained in 20 gal aquaria with treated water at 27 ± 1°C, on a 12:12 h light: dark cycle, and fed daily with live brine shrimp and Tetra-Min^® ^(Melle, Germany) flakes two times per day. All experiments were carried out in Georgetown, Guyana. Before starting each trial, all fish were fed to satiation.

Test females (n = 130) were periodically captured, individually housed in small 250 ml plastic containers to monitor their breeding status, and used within four days after parturition. We selected experimental females with standard lengths ≥ 20 mm (Mean ± SD: 26.48 ± 2.98 mm, Intervals 20.06 - 31.44 mm, n = 130). Considering the high levels of promiscuity in *P. parae *and that females breed year round [48,49], these females have likely bred at least once. Thus, we assumed that the selected standard length (≥ 20 mm) would be an indication that test females have been exposed to all five morphs under natural conditions. Another advantage in using post-partum females compared to naïve virgin females is that females mating preferences is shaped by previous experience of male phenotypes [60]; consequently, experienced females are more likely to posses better discrimination capabilities than naïve females [60,61]. Finally, previous mate choice experiments in *P. parae *have successfully recorded responses from experienced females towards all type of males [46,59,50]. Hence, for consistency, we followed the same criteria.

### Identification of morphs

Although the five *P. parae *morphs are easily identified by their patterns of coloration as adults, juvenile males resemble each other [46,47]. We used two methods to insure that the classification of males used for the experiments were accurate. First, we only used males with developed gonopodial hoods, which is a good indicator of sexually mature males in poeciliids [61-63]. Second, based on our observations of the development of fry in our laboratory, individuals express their distinct color patterns and have well differentiated gonopodium when they attain the body length of 13.9 - 14.5 mm (51-90 days in development; unpublished data). Similarly, Lindholm *et al*. [34] found that males reach sexual maturity when they attain a body length of 8.5 - 11.5 mm (n = 22) under laboratory conditions. Under experimental breeding conditions, growth of *P. parae *fry is slower and coloration is less intense compared to wild caught individuals, and so for the wild-caught males used in our study, we opted a more conservative, minimum standard length of 16 mm to reduce the possibility of choosing immature individuals. Having identified sexually-mature males, the classification of immaculata males was reliably determined.

### Matching body size for experimentation

Each test male was isolated two days before facing their assigned competitor and a corresponding female. Prior to each experiment, isolated test individuals were anaesthetized with MS-222 (3-aminobenzoic acid ethyl ester) and photographed using a camera (Canon EOS Rebel XTi 400 D digital, Japan). Each picture included a ruler as a metric reference. From each digital image, we measured the total length (tip of the upper jaw to tip of caudal fin) and standard length (tip of the upper jaw to the base of the caudal peduncle) of each fish. Body lengths were obtained by using Sigma Scan Pro^® ^v 5.0.0 (San Jose, CA, USA). With the exception of the small immaculata morph, test males were matched in length as much as possible (Table A1 in Additional file 1). Due to the fewer number of red and yellow males during our collections (see above), some individuals were used twice.

#### (a) Female mating preferences and male dominance

We assessed female mating preferences before ('pre-male competition') and after ('post-male competition') females observed male-male interactions. Each test female participated in a total of four trials with the same males in the following sequence: pre-competition mate choice → females observe male-male interactions (indirect) → post-competition mate choice → females and males directly interact (detailed below).

In the pre-male competition experiment, a random test female was presented with a dichotomous choice of males of two different morphs. Each female was only tested with one pair of males (in the sequence outlined above). All trials were staged in experimental 1.8 gal tanks (Figure A1, Additional file 1) partitioned in two zones by a transparent glass. The larger zone (60% of the total length of the tank) was used as the female test chamber. The smaller zone was further partitioned in two equal-sized compartments using a removable opaque plexiglass. The tank was supplied with a thin layer of gravel, filled to a depth of 14 cm with water, illuminated with a full spectrum light (70 cm above the tank), and covered on three sides with brown, kraft paper. The two small compartments were occupied by two randomly assigned males that were able to see the test female but not each other. The test female was placed in a removable opaque compartment situated in the centre of the female's test chamber. Males and female were acclimatized for 20 min before the start of each experiment. At the start of the experiment, the female compartment was lifted, and a Samsung Hi-8 SCL 860 camcorder positioned 30 cm away from the uncovered side of the tank recorded female mate choice for 10 min. Recordings started as soon as the test female approached the first male. From the video recording, we measured the amount of time (in seconds) that a female spent within a body length distance to the glass separating her from the males, and actively moving left and right while facing one of the two males. Periods in which a female remained within one body length of a particular male but did not reflect active inspection (i.e., not facing the male) were excluded. In *P. parae*, and poeciliids and other fishes, association time is a good index of female mating preferences [10,11,41-43,46,48-50,61,64,65].

Before running the post-male competition mate choice experiment, we transferred the three individuals (i.e., the two males and female) to a new 1.8 gal tank (Figure A1b), which was divided in two equal-sized sections by a clear glass. One section was occupied by the female and the other by the two competing males. The two males and female were allowed to acclimate for 10 min in individual, opaque removable compartments. At the start of the experiment, the partition separating the males was lifted, and we videotaped male-male interaction for 10 minutes, starting with the first male-male interaction. The test female was allowed to observe the two males through a transparent glass, with no physical contact. From the videotapes, we quantified all aggressive behaviours, but focused on three aggressive behaviours that occurred frequently: sparring, chasing, and attacks (see Table A2 for detailed definitions in Additional file 1). A male was declared dominant after the other male stopped approaching or interacting with the test female or opposing male, or assumed a headstand position whenever the dominant male approached (Table A2).

For the post-male competition experiment, both males and female were immediately returned to the first experimental tank, with the competing males assigned to the opposite compartment from the pre-male competition experiment. We quantified female mating preferences as in the pre-male competition experiment outlined above.

#### (b) Competition for access to females and mating success

To determine how male aggression directly affects copulation success, we allowed the same set of males and females to directly interact using an open-aquarium design. Males and the test female were transferred to individual compartments placed in the centre of a new 1.8 gal tank (Figure A1c in Additional file 1) and allowed to acclimate for 10 min. Individuals were released simultaneously, and were allowed to interact for 10 minutes, starting from the time of first interaction between males or a male and a female. All trials were video recorded, and we quantified all aggressive male-male interactions, aggressive male-female interactions, and number of successful copulations (as defined in [51]) that occurred during the 10 min trial).

Since other studies have shown that odours and pheromones influence mate choice and recognition in poeciliids (e.g., [66-68]), to reduce these effects, we used several aquaria, which allowed us to change the water between experiments.

#### (c) Analyses and statistics

Association time from pre- and post-male competition experiments were transformed to proportions for standardization purposes. We calculated preference for a particular male as the difference between the proportions of time spent between the two males. For instance, with M_A _and M_B _representing total proportion of time spent with male A or B, respectively, positive values of this index (M_A _- M_B_) would indicate a preference for male A and negative values a preference for male B. To determine whether there was a switch in female mating preferences after observing male-male interactions, we subtracted the post-male competition preference score from the pre-male competition preference score [i.e., (M_A _- M_B_) _2nd experiment _- (M_A _- M_B_) _1st experiment_]. We determined if females switched or enhanced their mate preferences between trials using a paired t-test. Preference scores were arcsine transformed to meet the assumptions of parametric tests [69].

We used two assays to measure the level of dominance of males of one particular morph over the other. First, we used the number of times individuals won against an opponent. For the dyadic contests over females, the outcomes (i.e., win or loss) were analysed with two-tailed exact binomial tests. Second, we also quantified the number of aggressive interactions between competitors, totalling then grouping the three types of the most frequent-observed aggressive behaviours [sparring (3%), chasings (21%), and attacks (71%)] into two categories: aggressions received and initiated. Both categories (aggressions received and initiated) were analysed using Kruskal-Wallis non-parametric ANOVAs. To determine which morphs were attacked less and attacked more, we ran multiple comparisons of mean ranks. We also performed Kruskal-Wallis non-parametric ANOVAs and multiple comparisons of mean ranks to analyse aggressions received and initiated during the open aquarium experiments. To compare whether there were differences in the number of attacks performed by males during direct and indirect interactions with females, we used a Mann-Whitney test. Finally, the difference in number of gained or lost copulations to the competitors was analysed with Wilcoxon matched pair tests. All data sets were tested for normality and analysed with STATISTICA^© ^ver. 7. StatSoft, Inc, 2007 OK, U.S.A.

## Authors' contributions

JLHG conceived the study, carried out the experiments, analysed the results and drafted the manuscript in partial fulfilment of a doctoral degree at Syracuse University, New York (USA). JACU contributed to the study design, supervised the study, edited and revised the manuscript critically. Both authors have read and approved the final manuscript.

## Supplementary Material

Additional file 1 **This file includes: Table A1 with additional information of the standard body lengths (mm) of males used during the experiments**. Table A2 presents a brief description of aggressive behaviours commonly displayed by males of *Poecilia parae*. Figure A1 presents a simplified view of the experimental settings. Click here for file

## References

[B1] Taborsky M Oliveira RF, Taborsky M, Brockmann JH Alternative reproductive tactics in fish Alternative reproductive tactics: an integrative approach 2008 Cambridge: Cambridge University Press 251 299 full_text

[B2] Gross MR Alternative reproductive strategies and tactics: diversity within sexes Trends Ecol Evol 1996 11 92 98 10.1016/0169-5347(96)81050-0 21237769

[B3] Oliveira RF Taborsky M Brockmann JH Alternative reproductive tactics: an integrative approach 2008 Cambridge: Cambridge University Press

[B4] Shuster SM Wade MJ Mating Systems and Strategies 2003 Princeton, New Jersey: Princeton University Press

[B5] Sinervo B Lively CM The rock-paper-scissors game and the evolution of alternative male strategies Nature 1996 380 240 243 10.1038/380240a0

[B6] Alonzo SH Sinervo B Mate choice games, context-dependent good genes, and genetic cycles in the side-blotched lizard, *Uta stansburiana* Behav Ecol Sociobiol 2001 49 176 186 10.1007/s002650000265

[B7] Lank DB Smith CM Hanotte O Burke T Cooke F Genetic polymorphism for alternative mating behaviour in lekking male ruff *Philomachus pugnax* Nature 1995 378 59 62 10.1038/378059a0

[B8] Pryke SR Griffith SC Red dominates black: Agonistic signalling among head morphs in the colour polymorphic Gouldian finch Proc R Soc Lond B 2006 273 949 957 10.1098/rspb.2005.3362 PMC1560236 16627280

[B9] Widemo F Alternative reproductive strategies in the ruff, *Philomachus pugnax*: a mixed ESS? Anim Behav 1998 56 329 336 10.1006/anbe.1998.0792 9787023

[B10] Kingston JJ Rosenthal GG Ryan MJ The role of sexual selection in maintaining a colour polymorphism in the pygmy swordtail, *Xiphophorus pygmaeus* Anim Behav 2003 65 735 743 10.1006/anbe.2003.2110

[B11] Morris MR Batra P Ryan MJ Male-male competition and access to females in the swordtail *Xiphophorus nigrensis* Copeia 1992 980 986 10.2307/1446627

[B12] Shuster SM Oliveira RF, Taborsky M, Brockmann JH The expression of crustacean mating strategies Alternative reproductive tactics: an integrative approach 2008 Cambridge: Cambridge University Press 224 250 full_text

[B13] Ryan MJ Pease CM Morris MR A genetic polymorphism in the swordtail *Xiphophorus nigrensis*: testing the prediction of equal fitnesses Am Nat 1992 139 21 31 10.1086/285311

[B14] Kirkpatrick M Ryan MJ The evolution of mating preferences and the paradox of the lek Nature 1991 350 33 38 10.1038/350033a0

[B15] Kokko H Brooks R Jennions MD Morley J The evolution of mate choice and mating biases Proc R Soc Lond 2003 270 653 664 10.1098/rspb.2002.2235 PMC1691281 12769467

[B16] Blows MW Hoffmann AA A reassessment of genetic limits to evolutionary change Ecology 2005 86 1371 1384 10.1890/04-1209

[B17] Tomkins JL Radwan J Kotiaho JS Tregenza T Genic capture and resolving the lek paradox Trends Ecol Evol 2004 19 323 328 10.1016/j.tree.2004.03.029 16701278

[B18] Brooks R Endler JA Direct and indirect sexual selection and quantitative genetics of male traits in guppies (*Poecilia reticulata*) Evolution 2001 55 1002 1015 10.1554/0014-3820(2001)055[1002:DAISSA]2.0.CO;2 11430637

[B19] Candolin U Opposing selection on a sexually dimorphic trait through female choice and male competition in a water boatman Evolution 2004 58 1861 1864 15446439 10.1111/j.0014-3820.2004.tb00470.x

[B20] Moore AJ Moore PJ Balancing sexual selection through opposing mate choice and male competition Proc R Soc Lond 1999 266 711 716 10.1098/rspb.1999.0694

[B21] Galeotti P Rubolini D Dunn PO Fasola M Colour polymorphism in birds: causes and functions J Evol Biol 2003 16 635 646 10.1046/j.1420-9101.2003.00569.x 14632227

[B22] Roulin A The evolution, maintenance and adaptive function of genetic colour polymorphism in birds Biol Rev 2004 79 815 848 10.1017/S1464793104006487 15682872

[B23] Ra'Anan Z Sagi A Alternative mating strategies in male morphotypes of the freshwater prawn *Macrobrachium rosenbergii *(De Man) Biol Bull 1985 169 592 601

[B24] Gadgil M Male Dimorphism as a Consequence of Sexual Selection Am Nat 1972 106 574 580 10.1086/282797

[B25] Barlow GW Do gold Midas cichlid fish win fights because of their color, or because they lack normal coloration? A logistic solution Behav Ecol Sociobiol 1983 13 197 204 10.1007/BF00299923

[B26] Morris MR Tudor MS Dubois NS Sexually selected signal attracted females before deterring aggression in rival males Anim Behav 2007 74 1189 1197 10.1016/j.anbehav.2007.01.019

[B27] Healey M Uller T Olsson M Seeing red: morph-specific contest success and survival rates in a colour-polymorphic agamid lizard Anim Behav 2007 74 337 341 10.1016/j.anbehav.2006.09.017

[B28] Tuttle EM Alternative reproductive strategies in the white-throated sparrow: behavioral and genetic evidence Behav Ecol 2003 14 425 432 10.1093/beheco/14.3.425

[B29] Mulder RA Ramiarison R Emahalala RE Ontogeny of male plumage dichromatism in Madagascar paradise flycatchers *Terpsiphone mutata* J Avian Biol 2002 33 342 348 10.1034/j.1600-048X.2002.02888.x

[B30] Korzan WJ Fernald RD Territorial male color predicts agonistic behavior of conspecifics in a color polymorphic species Behav Ecol 2007 18 318 323 10.1093/beheco/arl093

[B31] Sinervo B Calsbeek R The Developmental, Physiological, Neural, and Genetical Causes and Consequences of Frequency-Dependent Selection in the Wild Annu Rev Ecol Syst 2006 37 581 610 10.1146/annurev.ecolsys.37.091305.110128

[B32] Hori M Frequency-dependent natural selection in the handedness of scale-eating cichlid fish Science 1993 260 216 219 10.1126/science.260.5105.216 17807183

[B33] Losey JE Harmon J Ballantyne F Brown C A polymorphism maintained by opposite patterns of parasitism and predation Nature 1997 388 269 272 10.1038/40849

[B34] Munday P Eyre P Jones G Ecological mechanisms for coexistence of colour polymorphism in a coral-reef fish: an experimental evaluation Oecologia 2003 137 519 526 10.1007/s00442-003-1356-7 13680346

[B35] Gray SM McKinnon JS Linking color polymorphism maintenance and speciation Trends Ecol Evol 2007 22 71 79 10.1016/j.tree.2006.10.005 17055107

[B36] Ayala FJ Campbell CA Frequency-Dependent Selection Annu Rev Ecol Syst 1974 5 115 138 10.1146/annurev.es.05.110174.000555

[B37] Andersson MB Sexual selection 1994 Princeton, New Jersey: Princeton University Press

[B38] Qvarnström A Forsgren E Should females prefer dominant males? Trends Ecol Evol 1998 13 498 501 10.1016/s0169-5347(98)01513-4 21238407

[B39] Wong BBM Candolin U How is female mate choice affected by male competition? Biol Rev 2005 80 559 571 10.1017/S1464793105006809 16221329

[B40] Kodric-Brown A Male dominance can enhance mating success in guppies Anim Behav 1992 44 165 167 10.1016/S0003-3472(05)80766-3

[B41] Kodric-Brown A Female choice of multiple male criteria in guppies: Interacting effects of dominance, coloration and courtship Behav Ecol Sociobiol 1993 32 415 420

[B42] Casalini M Agbali M Reichard M Konečná M Bryjová A Smith C Male dominance, female mate choice, and intersexual conflict in the rose bitterling (*Rhodeus ocellatus*) Evolution 2009 63 366 376 10.1111/j.1558-5646.2008.00555.x 19154367

[B43] Reichard M Bryja J Ondracková M Dávidová M Kaniewska P Smith C Sexual selection for male dominance reduces opportunities for female mate choice *in the European b*itt*erling (Rhodeus sericeus*) Mol Ecol 2005 14 5 1533 1542 10.1111/j.1365-294X.2005.02534.x 15813791

[B44] Alonzo SH Oliveira RF, Taborsky M, Brockmann JH Conflict *between the sexe*s a*nd altern*ative reproductive tactics within a sex Alternative reproductive tactics: an integrative approach 2008 Cambridge: Cambridge University Press 435 450 full_text

[B45] Wade MJ Shuster SM Sexual Selection: Harem size and the variance in male reproductive success Am Nat 2004 164 E83 E89 10.1086/424531 15459886

[B46] Lindholm AK Brooks R Breden F Extreme polymorphism in a Y-linked sexually selected trait Heredity 2004 92 156 162 10.1038/sj.hdy.6800386 14735138

[B47] Lindholm AK Hunt J Brooks R Where do all the maternal effects go? Variation in offspring body size through ontogeny in the live-bearing fish *Poecilia parae* Biol Lett 2006 2 586 589 10.1098/rsbl.2006.0546 17148295 PMC1833979

[B48] Liley NR Ethological Isolating Mechanisms in Four Sympatric Species of Poeciliid Fishes Behaviour 1966 III 197

[B49] Bourne GR Breden F Allen TC Females prefer carotenoid colored males as mates in the pentamorphic livebearing fish, *Poecilia parae* Naturwissenschaften 2003 90 402 405 10.1007/s00114-003-0444-1 14504782

[B50] Hurtado-Gonzales JL Baldassarre DT Uy JAC Interaction between female mating preferences and predation may explain the maintenance of rare males in the pentamorphic fish *Poecilia parae* J Evol Biol 2010 23 1293 1301 10.1111/j.1420-9101.2010.01995.x 20456563

[B51] Hurtado-Gonzales JL Uy JAC Alternative mating strategies may favour the persistence of a genetically based colour polymorphism in a pentamorphic fish Anim Behav 2009 77 1187 1194 10.1016/j.anbehav.2008.12.032

[B52] Jukema J Piersma T Permanent female mimics in a lekking shorebird Biol Lett 2006 2 161 164 10.1098/rsbl.2005.0416 17148353 PMC1618908

[B53] Shuster SM Wade MJ Equal mating success among male reproductive strategies in a marine isopod Nature 1991 350 608 610 10.1038/350608a0

[B54] Smith C Reichard M Females solicit sneakers to improve fertilization success in the bitterling fish (*Rhodeus sericeus*) Proc R Soc Lond 2005 272 1683 1688 10.1098/rspb.2005.3140 PMC1559859 16087423

[B55] Pilastro A Simonato M Bisazza A Evans JP Cryptic female preference for colorful males in guppies Evolution 2004 58 665 669 15119451

[B56] Sinervo B Bleay C Adamopoulou C Social causes of correlational selection and the resolution of a heritable throat color polymorphism in a lizard Evolution 2001 55 2040 2052 11761064 10.1111/j.0014-3820.2001.tb01320.x

[B57] Fuller RC Travis J Genetics, lighting environment, and heritable responses to lighting environment affect male color morph expression in bluefin killifish, *Lucania goodei* Evolution 2004 58 1086 1098 15212389 10.1111/j.0014-3820.2004.tb00442.x

[B58] Chunco A McKinnon JS Servedio MR Microhabitat variation and sexual selection can maintain male colour polymorphisms Evolution 2007 61 2504 2515 10.1111/j.1558-5646.2007.00213.x 17725638

[B59] Gray SM Dill LM Tantu FY Loew ER Herder F McKinnon JS Environment-contingent sexual selection in a colour polymorphic fish Proc R Soc Lond 2008 275 1785 1791 10.1098/rspb.2008.0283 PMC2587793 18445554

[B60] Breden F Novinger D Schubert A The effect of experience on mate choice in the Trinidad guppy, *Poecilia reticulata* Environ Biol Fishes 1995 42 323 328 10.1007/BF00004926

[B61] Houde AE Sex, color, and mate choice in guppies 1997 Princeton, New Jersey: Princeton University Press

[B62] Rosen DE Gordon M Functional anatomy and evolution of male genitalia in poeciliid fishes Zoologica 1953 38 1 47

[B63] Meffe GK Snelson FF Ecology and evolution of livebearing fishes (Poeciliidae) 1989 Englewood Cliffs, New Jersey: Prentice Hall 10.1126/science.248.4954.502-a 17815603

[B64] Cummings M Mollaghan D Repeatability and consistency of female preference behaviours in a northern swordtail, *Xiphophorus nigrensis* Anim Behav 2006 72 217 224 10.1016/j.anbehav.2006.01.009

[B65] Young M Simmons L Evans J Pre- and post-mating sexual selection both favor large males in a rainbowfish Behav Ecol Sociobiol 64 915 925 10.1007/s00265-010-0906-3

[B66] Crapon de Caprona MD Ryan MJ Conspecific mate recognition in swordtails, *Xiphophorus nigrensis *and *X. pygmaeus *(Poeciliidae): olfactory and visual cues Anim Behav 1990 39 290 296 10.1016/S0003-3472(05)80873-5

[B67] Decaprona MDC Ryan MJ Conspecific mate recognition in swordtails, *Xiphophorus nigrensis *and *X. Pygmaeus *(Poeciliidae): olfactory and visual cues Anim Behav 1990 39 290 296 10.1016/S0003-3472(05)80873-5

[B68] Crow RT Liley NR A sexual pheromone in the guppy, *Poecilia reticulata *(Peters) Can J Zool 1979 57 184 188 10.1139/z79-016

[B69] Sokal RR Rohlf FJ Biometry: the principles and practice of statistics in biological research 1995 3 New York: Freeman

