# Supplementary material for: Intrasexual competition facilitates the evolution of alternative mating strategies in a colour polymorphic fish
Source: BMC Evol Biol. 2010 Dec 23;10:391. doi: 10.1186/1471-2148-10-391 (PMC3017046; doi:10.1186/1471-2148-10-391)

Table A1. Standard body length (mm) of *P. parae* males that were used in male-male competition trials. Columns to the left provide the descriptive statistics (Mean ± SD, minimum and maximum) for males from one morph, while adjacent columns to the right provide the descriptive statistics for the corresponding males used from a second morph **(**I = immaculata, P = parae, B = blue, R = red, Y= yellow). Sample sizes for each set of trials are provided (*n)*. The final two columns to the right provide the mean difference (± SD) between competing males (M_A-M_B). Negative values indicate that male 2 (M_B) was larger on average than male 1 (M_A).

M_A	Mean	SD	Min	Max	M_B	Mean	SD	Min	Max	n	M_A - M_B	SD
I	16.831	0.395	16.168	17.401	P	19.272	0.658	18.35	20.38	10	-2.440	0.296
	16.684	0.42	16.033	17.387	B	17.133	0.273	16.625	17.401	10	-0.449	0.19
	16.782	0.482	16.059	17.48	R	18.607	0.151	18.267	18.813	10	-1.825	0.35
	16.82	0.553	16.066	17.455	Y	18.143	0.254	17.739	18.568	10	-1.322	0.331
P	18.951	0.891	16.833	19.876	B	18.864	0.92	17.56	20.161	15	0.087	0.364
	19.717	0.465	19.075	20.342	R	19.879	0.467	19.442	20.9	15	0.161	0.196
	19.396	1.042	16.457	20.257	Y	19.731	0.377	19.334	20.735	15	-0.335	0.868
B	19.928	0.685	19.038	20.972	R	19.685	0.662	18.877	20.9	15	0.242	0.209
	19.24	0.737	18.437	20.083	Y	19.308	0.629	18.705	20.735	15	-0.068	0.447
R	18.924	0.409	18.267	19.418	Y	18.78	0.627	17.739	19.869	15	0.144	0.326

Table A2. Aggressive behaviours observed during male-male interactions in *Poecilia parae*. Modified from the terminology and description Liley (1966) offered for *P. reticulata* (the guppy).

Sparring: This behaviour is mostly initiated by a single male (challenger). The challenger will approach to the other male presenting one of its flanks with the dorsal and ventral fins fully extended and the body slightly arched towards the side facing the flank of the opponent. The body of the challenger will start quivering as an invitation to initiate the sparring. If the opponent accepts the challenge, it will immediately adopt an anti-parallel or parallel position to that of the challenger. During this period, the body and fin colorations become intense. Once both males are placed in their position, they will keep their body quivering and performing circular motions and moving upwards. During circling, both males would keep their initial posture. This behaviour can last between 2 – 45 seconds and can be repeated several times if none of the males are willing to retreat.

Tail slapping: This behaviour follows sparring and can be initiated by either the challenger or the opponent. The slap would be either directed towards the flank or head region of the opponent. If dominance has not yet been determined, males will continue tail slapping for short periods of time (2 - 4 seconds), and males will re-group in sparring position. However, if one of the males retreats, the tail slapping will be followed by an attack.

Chasing: the dominant male will go behind the loser male performing consecutive attacks. Chasing usually has an average of three attacks and/or chase and ends with male's complete submission by head standing.

Attack: attacks are comprised by nibbles directed towards the flank of the other individual. However, the winner can nibble and keep attacking until the opponents flee, or can give a warning nibble and will resume their behaviour (e.g., eating or courting).

Escape: the male losing the male-male interaction usually flee away of the dominant male. If the male decides to remain close to the dominant male, attacks can continue and followed by chasing.

Headstands: to stop chase or attacks, the subordinate male will tilt his body vertically forming and angle > 45° to the substrate.

Blocking: this behaviour is commonly observed after a male assumes the dominant role. The male will block access of other males to the female by not allowing other males to approach to the females. The dominant male will use his body to intercept the approach of the other males.

Figure A1. Experimental tanks. (a) Tank use for the female choice trials. The glass separating both males is a fixed non transparent barrier. (b) Tank use to determine aggressive behaviours between males, with a female observing the interactions through a transparent glass barrier. (c) Tank use to determine aggressive behaviours between males with the direct interaction of a female.

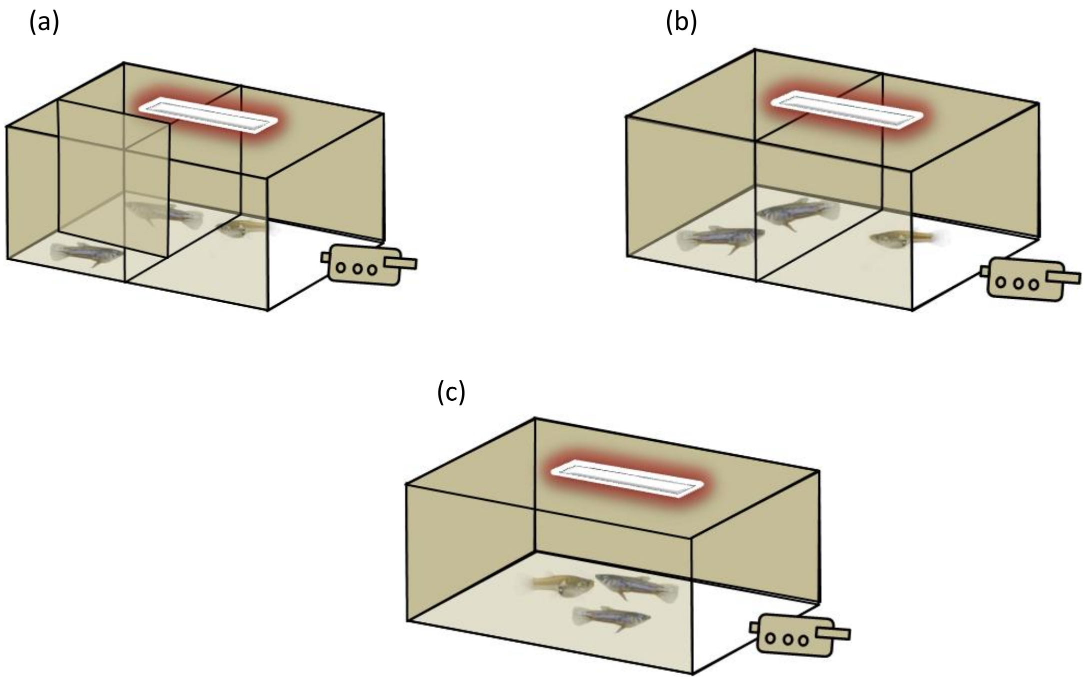

Supplement: Additional file 1 — This file includes: Table A1 with additional information of the standard body lengths (mm) of males used during the experiments. Table A2 presents a brief description of aggressive behaviours commonly displayed by males of Poecilia parae. Figure A1 presents a simplified view of the experimental settings. [file 1471-2148-10-391-S1.PDF]
